# Assessment of fluid responsiveness during prone position in ards. a validation study

**DOI:** 10.1186/2197-425X-3-S1-A591

**Published:** 2015-10-01

**Authors:** J-C Richard, H Yonis, F Bayle, F Gobert, R Taponnier, V Leray, C Guérin

**Affiliations:** Hospices Civils de Lyon, Service de Réanimation Médicale - Hôpital de la Croix-Rousse, Lyon, France; Université de Lyon - Université Lyon I, Lyon, France; CREATIS INSERM 1044 CNRS 5220, Villeurbanne, France; IMRB INSERM 955, Equipe 13, Creteil, France

## Introduction

Predicting fluid responsiveness is of paramount importance to avoid unnecessary fluid administration in ARDS patients, since a positive fluid balance is associated with ARDS mortality [1]. Several tests with high reliability to predict fluid responsiveness are now available, but none have been validated in the prone position (PP) in ARDS patients, while this treatment is now a cornerstone of the therapeutic armamentarium of severe ARDS [2].

## Objectives

To evaluate the diagnostic performance of three methods to predict fluid responsiveness in PP: cardiac index variation during the Trendelenburg position and an end-expiratory occlusion, and pulse pressure variation after increasing tidal volume (VT) to 8 ml.kg^-1^ predicted body weight (PBW).

## Methods

This study is a prospective single-center study, performed on ARDS patients in PP, monitored with the PiCCO device, and with acute circulatory failure. Patients were studied at baseline with bed angulation 13°, during a 1-min postural change in the Trendelenburg position with bed angulation -13°, during a 1 min increase of VT to 8 ml.kg^-1^ PBW, during a 15-s end-expiratory occlusion and after IV infusion of 500 ml crystalloids. Patients were returned to baseline settings after each intervention. Fluid responsiveness was deemed present if cardiac index assessed by thermodilution increased by at least 15% after fluid administration.

## Results

19 patients (SAPS II 56 (48-62)) were included at the time of abstract submission. VT was 6.0 (5.9-6.2) ml.kg^-1^ PBW, PaO_2_/FiO_2_ ratio 158 mm Hg (116-201), PEEP 8 (6-10) cm H_2_O, and plateau pressure 23 (20-27) cm H_2_O. 7 patients (37%) were deemed fluid-responsive after fluid administration. The area under ROC curve of the pulse contour derived cardiac index during the Trendelenburg maneuver and the end-expiratory occlusion test were 0.91 (95% CI: 0.77-1) and 0.45 (95% CI: 0.17-0.73), respectively. An increase in cardiac index ≥ 8% during the Trendelenburg maneuver enable to diagnose fluid responsiveness with a sensitivity of 71% (95% CI: 43-100%), and a specificity of 100% (95% CI: 100-100%). Cardiac arrhythmia were present at baseline in 11 patients (58%), which were therefore excluded from pulse pressure variation analysis. The area under ROC curve of pulse pressure variation during VT increase to 8 ml.kg^-1^PBW was 0.50 (95% CI: 0.04-0.96).

## Conclusions

Cardiac output measurement during a Trendelenburg maneuver is a reliable method to assess fluid responsiveness in ARDS patients in the prone position.

## Trial Registration

Clinical trial registered with www.clinicaltrials.gov (NCT01965574).
